# Variations in the phenological patterns of a caddisfly inhabiting the same mountain massifs: Life‐history differences in different altitudinal zones

**DOI:** 10.1002/ece3.11428

**Published:** 2024-06-06

**Authors:** Hirohisa Suzuki, Masaki Takenaka, Koji Tojo

**Affiliations:** ^1^ Division of Mountain and Environmental Science, Interdisciplinary Graduate School of Science and Technology Shinshu University Nagano Japan; ^2^ Department of Biology, Faculty of Science Shinshu University Nagano Japan; ^3^ Institute of Mountain Science Shinshu University Nagano Japan

**Keywords:** cold‐adapted species, imaginal diapause, Limnephilidae, mountain ecology, sky island, Trichoptera

## Abstract

Organisms inhabiting mountainous regions can experience large vertical environmental changes, and show different ecological characteristics between altitudes, thus facilitating allopatric fragmentation even in geographically close populations. This study compared the life‐history patterns of a species of limnephilid caddisfly, *Asynarchus sachalinensis*, in several genetically differentiated populations between alpine and sub‐alpine zones in a temperate mountainous region. We showed that in the sub‐alpine populations, larval development started earlier with increasing water temperature in spring, and adult emergence was also earlier. The occurrence of adults was extremely low in mid‐summer, probably due to summer diapause, followed by a larger number of ovary‐developed females in autumn. On the other hand, in the alpine zone, increasing water temperature was delayed compared to the sub‐alpine zone, and larval development occurred from early to mid‐summer. Adult emergence and ovary‐developed individuals were concentrated in mid‐summer. Hence, summer diapause was not observed. These results indicated life‐history differences between genetically differentiated populations at different altitudes. As the timing of adult occurrence and ovarian developmental patterns differ between populations at different altitudes, it is possible that reproductive isolation is facilitated or maintained between populations.

## Graphical abstract

The phenological variations of an aquatic insect within the same temperate mountain massif were compared between alpine and sub‐alpine populations. Differences in phenological variations due to environmental differences were indicated between populations from different altitudinal zones.

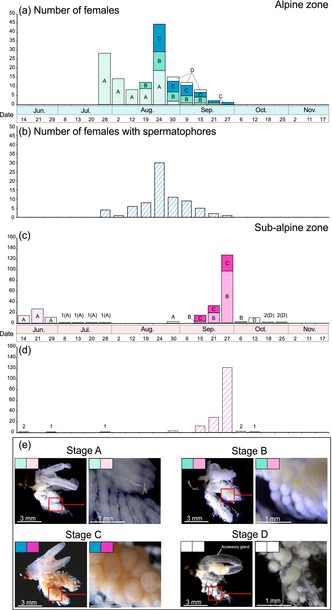

## 1 INTRODUCTION

When geographically isolated sub‐populations adapt to their different environments and accumulate their own unique set of evolutionary adaptation, resulting in ecological differentiation, reproductive isolation may eventually be established and maintained, even after secondary contact has occurred (Albertson et al., 1999; Rundle & Nosil, 2005; Schluter, 2001; Seehausen et al., 1997). It is well known that life‐history patterns often differ between populations inhabiting different altitudinal zones of the same mountain range (McCarty et al., 2022; Nebeker, 1971). In addition, daylength, nutritional conditions and water temperature have been shown to be particularly important factors regulating life‐history stages (postembryonic development) of aquatic insects that spend their larval stages in water (Bonacina et al., 2023; Füreder et al., 2005; Hayashi, 1990; Sweeney, 1984; Vannote & Sweeney, 1980; Ward & Stanford, 1982). Especially, in the case of lentic waters at high altitudes, the source of water in waterbodies is often dependent on snow cover, and the pattern of water temperature fluctuations varies considerably from place to place depending on the amount of snow cover, the location of the snow cover, and the timing of snow melt (Nebeker, 1971). Therefore, habitat differentiation is high, and life‐history differences are correspondingly highly likely to occur between habitats (Butterfield, 1996; Goffová et al., 2015; McCulloch & Waters, 2018; Ward & Stanford, 1982). In addition, if there are differences in the reproductive timing of each population, reproductive isolation between populations drives speciation and/or intra‐specific differentiation (Rundle & Nosil, 2005; Schluter, 2001).

For several cold‐adapted species of limnephilid caddisflies, which inhabit lentic waters such as ponds and springs, it is known that they emerge with undeveloped ovaries and undergo diapause (i.e., “imaginal diapause”) in caves and tree canopies until the temperature drops and/or water levels recover (Crichton, 1971; Denis, 1978; Novak & Sehnal, 1963; Salavert et al., 2008; Svensson, 1972). However, the occurrence and period of such imaginal diapause vary between species and populations (Crichton, 1971; Denis, 1978; Gíslason, 1978; Svensson, 1972). Factors contributing to differences in the occurrence and periods of imaginal diapause are explained by, for example, temperature and/or photoperiod (Alekseev & Starobogatov, 1996; Denis, 1978). One of the previous studies have shown experimentally that *Limnephilus rhombicus*, a species of limnephilid caddisflies, is prompted to induce and terminate “imaginal diapause” based on the daylength experienced during the 5th instar larval period (Denis, 1978). Differences in developmental timing caused by differences in water temperature may also occur, and along with differences in the daylength experienced by 5th instar larvae, these factors contribute to differentiation between populations and the occurrence of imaginal diapause (Bouchard & Ferrington, 2009; Finn et al., 2022; Hogue & Hawkins, 1991). If there is differentiation in the timing of the adult (flying stage), it results in differentiation in the timing of the reproductive stage. Therefore, gene flow between populations is restricted, and such gene flow and genetic differentiation are facilitated. In low‐latitude temperate mountainous regions, it may result in differences in the life histories of the species inhabiting such areas because of large environmental differences between altitudinal zones (Laiolo & Obeso, 2017; Shah et al., 2017). However, there have been few studies comparing the life histories of the same species between altitudinal zones in such mountainous regions.

**FIGURE 1:**
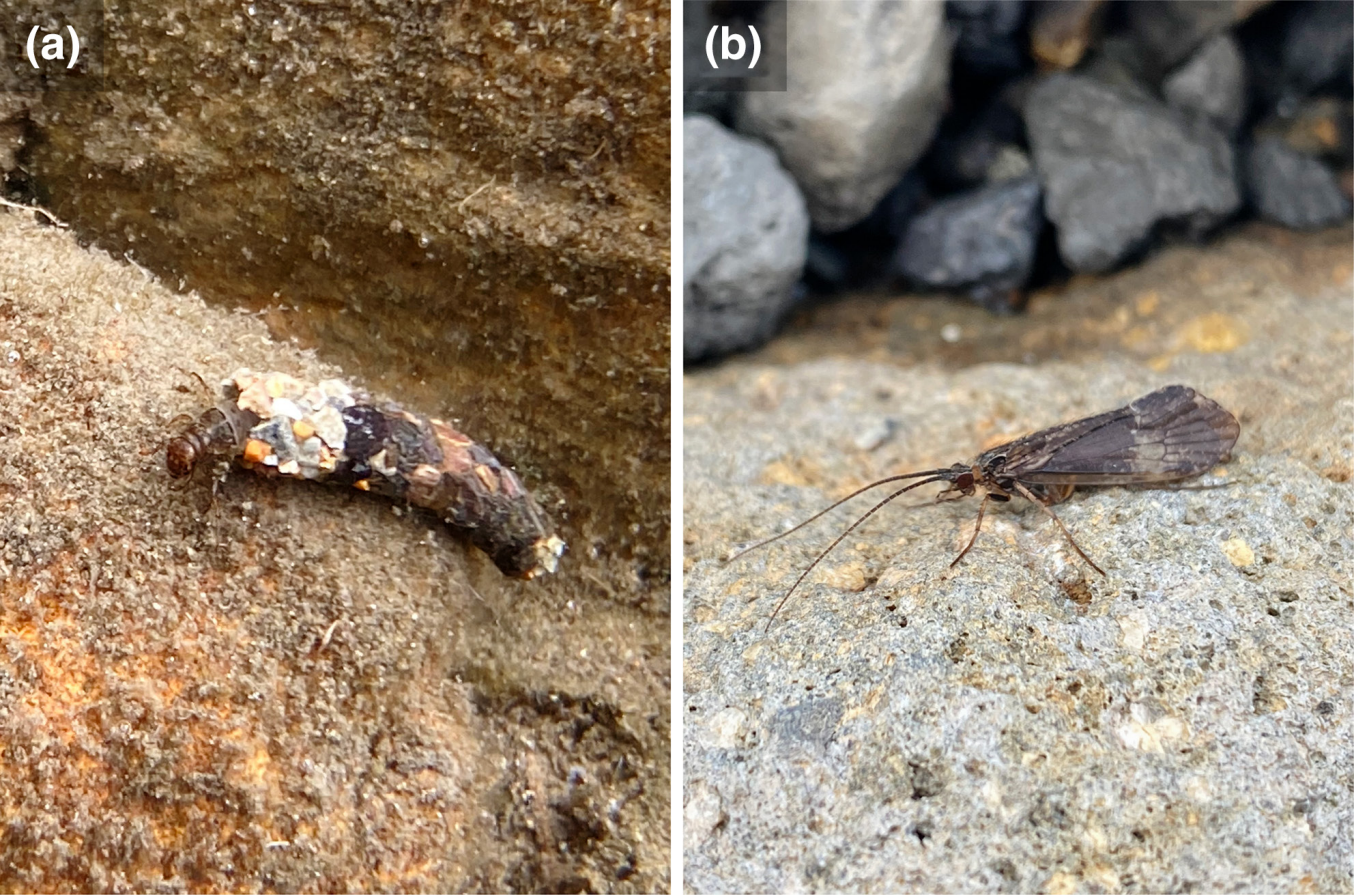
(a) *Asynarchus sachalinensis* 5th instar larva crawling bottom of the water body (AL2). (b) A female adult (AL1).

The target species of our study, *Asynarchus sachalinensis* Martinov 1914 (Figure 1), is a species of Limnephilidae with distributions in Sakhalin, Hokkaido and central to northern regions of Honshu. *Asynarchus sachalinensis* inhabits cool temperature water bodies (e.g., ponds and springs) (Ito, 2008; Nozaki, 2002), and also high‐altitude wetlands and caldera lakes of alpine zones (Nozaki, 2002; Suzuki et al., 2024). A previous life‐history study of populations distributed in Hokkaido showed that larvae grow rapidly and synchronously in spring (April to May), emerge in early summer (in June), enter imaginal diapause in mid‐summer (July to August), and lay eggs in autumn (September to October) (Ito, 2008). We showed in a previous study that this caddisfly has a univoltine lifecycle (Ito, 2008). In addition, our own previous study showed that *A. sachalinensis* collected from the entire distributional area including this study area (i.e., Mt. Norikura‐dake, alpine and sub‐alpine zones) consists of two genetically differentiated intra‐specific clades based on mitochondrial DNA sequences (Suzuki et al., 2024). For these intra‐specific clades, a relationship between the types of habitats and genetic differentiation was indicated (Suzuki et al., 2024). One of the genetic clades inhabits low lands while the other, inhabits high‐altitudes area. Habitats at high altitudes are often isolated and discontinuous with nearby lowlands (e.g., basins). With such a pattern, the loss of population connectivity restricts chances for gene flow between populations (Knowles, 2001; Mikami et al., 2023; Pauls et al., 2006; Theissinger et al., 2013; Uscanga et al., 2021). In addition, even if populations are geographically close to each other, gene flow between populations is highly restricted, such that populations often show distinct genetic structures from each other (Mikami et al., 2023; Theissinger et al., 2013; Uscanga et al., 2021).

These considerations led us to study the relationship between habitat and life history in six populations (three populations inhabiting alpine zones and three populations inhabiting sub‐alpine zones) of a cold‐adapted species on Mt. Norikura‐dake, one of the southernmost mountain ranges of the species' distribution.

## 2 MATERIALS AND METHODS

### 2.1 Study sites and a targeted species

**FIGURE 2:**
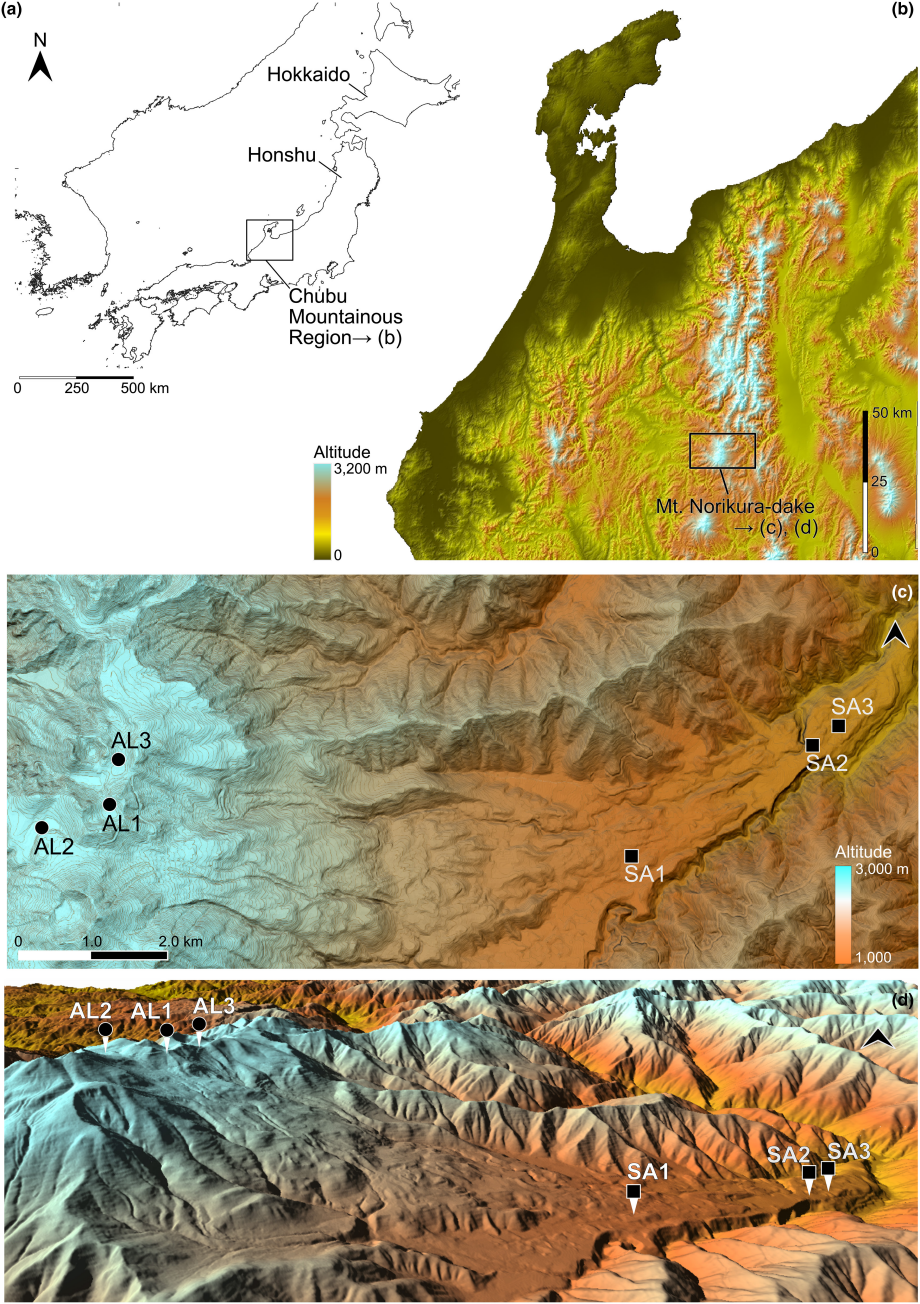
Schematic maps of the study sites. (a, b) Mt. Norikura‐dake (36.106 N, 137.553 E, 3026 m asl.) where the study sites were located is in the Chubu Mountainous Region in central Honshu. (c, d) A plain view map and a bird's‐eye view map of Mt. Norikura‐dake. Study sites AL1–3 are located in the alpine zone, and sites SA1–3 sites are in the sub‐alpine zone of Mt. Norikura‐dake.

Mt. Norikura‐dake (36.106 N, 137.554 E, 3026 m asl.), where the six water bodies studied are located, is a cluster of active volcanic calderas in the Chubu Mountainous Region of Honshu in the Japanese Archipelago (Shimizu, 1990) (Figure 2). The results of our previous study established that the water bodies of Mt. Norikura‐dake are inhabited by large numbers of the target species *Asynarchus sachalinensis* at two different altitude zones (Suzuki et al., 2024). Previous studies indicated the forest limit to be at approximately 2500 m alt. on the east side of Mt. Norikura‐dake (Miyajima & Takahashi, 2007; Takahashi et al., 2003). Of the two groups of study sites, one is located between approximately 1200 m and 1500 m asl. (SA1: Lake Ohmagari‐ike, 1424 m asl.; SA2: Lake Chidori‐ike, 1252 m asl.; SA3: Miyanohara wetland, 1227 m asl.), within the sub‐alpine zone where deciduous broad‐leaved trees dominate the vegetation (Miyajima & Takahashi, 2007; Miyajima et al., 2007). The other study sites are at approximately 2700 m asl. (AL1: Lake Kiezuga‐ike, 2737 m asl.; AL2: Lake Gono‐ike, 2725 m asl.; AL3: Lake Tsuruga‐ike, 2703 m asl.), located within the alpine zone where the alpine dwarf pine tree dominates (Miyajima & Takahashi, 2007; Miyajima et al., 2007). Dead broad‐leaves were found on the bottom of water bodies in the sub‐alpine zone and dead shrub cores (i.e., of alpine dwarf pine trees) were found on the bottoms of water bodies in the alpine zone. The water bodies of all study sites were lentic. The area (m^2^) and circumference (m) of the water bodies were calculated based on 5 m mesh DEM maps from GSI (Geospatial Information Authority of Japan: https://www.gsi.go.jp/). The information on the study sites is shown in Table 1, Figures 2c,d and S1.

**TABLE 1:** Study site information list.

Study sites							
Locality ID	Locality name	Latitude (*N*)	Longitude (E)	Altitude (m asl.)	Perimeter (m)	Area (m^2^)	The deadleaves of the waterbody bottom
AL 1	Lake Kiezuga‐ike, Mt. Norikura‐dake, Nyukawa, Takayama, Gifu	36.1199	137.5549	2,737	290	4,512	Alpine dwarf pine (i.e., *Pinus pumila*)
AL 2	Lake Gono‐ike, Mt. Norikura‐dake, Nyukawa, Takayama, Gifu	36.1180	137.5471	2,725	330	6,840	Alpine dwarf pine (i.e., *Pinus pumila*)
AL 3	Lake Tsuruga‐ike, Mt. Norikura‐dake, Nyukawa, Takayama, Gifu	36.1252	137.5557	2,703	275	3,910	Alpine dwarf pine (i.e., *Pinus pumila*)
SA 1	Lake Ohmagari‐ike, Norikura‐highland, Azumi, Matsumoto, Nagano	36.1133	137.6326	1,424	183	1,719	Deciduous broad‐leaved tree
SA 2	Lake Chidori‐ike, Norikura‐highland, Azumi, Matsumoto, Nagano	36.1274	137.6594	1,252	216	2,807	Deciduous broad‐leaved tree
SA 3	Miyanohara‐wetland, Norikura‐highland, Azumi, Matsumoto, Nagano	36.1293	137.6624	1,227	145	1,272	Deciduous broad‐leaved tree

Phylogenetic analyses conducted in our previous study using the mitochondrial DNA (mtDNA) combined sequences of the COI (648 bp), and the 16S rRNA (417 bp) regions showed that the respective populations of *A. sachalinensis* within these two altitudinal zones belong to distinct genetic clades, and that populations within the same altitudinal zone are genetically close (Figure S2: Suzuki et al., 2024).

### 2.2 Sampling design for assessment of life‐history differentiation between the alpine and sub‐alpine zone populations

Weekly larval developmental stage surveys were conducted to compare the life histories of *A. sachalinensis* in those two different altitude zones of Mt. Norikura‐dake (conducted surveys every week 32 times, from April 10 to November 17, 2022). In order to compare the larval period of *A. sachalinensis* inhabiting alpine and sub‐alpine zones, larvae and pupae were collected quantitatively at each study site and on each sampling date. For collection of larvae, the same researcher selected five sampling points at each study site randomly and on each sampling date and collected by hand all larvae in quadrats (0.3 × 0.3 m). To identify larvae instar, image analysis software Image J 1.53 k (Schneider et al., 2012) was used to measure head width. Head width measurements and histogram construction using head width data were based on methodology used in a previous study of the life cycle of *A. sachalinensis* (Ito, 2008). Multiple histogram peaks were detected and the number of peaks was used to identify the instar stage number.

Adults were collected using light traps with 3 W ultraviolet LEDs (Figure S3) from sunset to sunrise at the same locations (two sampling points) at each study site. All adult specimens collected that had fallen onto the surface of the water in the light trap trays were counted while distinguishing between males and females. Adult emergence surveys were continued on two consecutive days at weekly intervals in each altitudinal zone until no adults were observed at all (the start observation date for the alpine zone was July 8, last observation was October 18; the start observation date for the sub‐alpine zone was June 14, last observation date was November 17). However, one of the scheduled surveys in the alpine zone was not conducted due to bad weather conditions (October 6).

The degree of ovarian development was classified into four categories—Stages A–D, based on Stage A: a stage with undeveloped reproductive organs (non‐egg‐maturation stage); Stage B: a stage in which yolk accumulation is insufficient (egg‐maturation stage); Stage C: a stage in which yolk accumulation is sufficient, with fully mature eggs (egg‐maturation stage); Stage D: a stage in which the ovary regresses after oviposition (post‐oviposition stage). Furthermore, females with spermatophores in the bursa copulatrix were counted with reference to Ito (2020).

To measure and compare environmental factors at each study site, EC (electrical conductivity), DO (dissolved oxygen), and pH were measured using an EC meter (Cyberscan CON 400, Nijkerk, Netherlands), a DO meter (MM‐41DP, TOA DKK, Tokyo, Japan) and a pH meter (392R, AS ONE, Osaka, Japan). Surface water was collected at five points (from which larvae were collected) at each study site and on each sampling date, and each sample was measured for EC, DO, and pH on site. The averages of the measured data were used as the representative data recordings for each study site and sampling date. To assess the canopy openness rate, digital photographs were taken with a camera equipped with a fisheye lens (EX‐FR200, CASIO, Tokyo, Japan) at six points around the ponds at each study site every month. Each photo was image‐analyzed using CanopOn2 software (http://takenaka‐akio.org/etc/canopon2/), and then averaged. In the image analyses, 10% of the image margin was removed in order to eliminate the influence of artificial objects and/or background mountains. In adult emergence surveys, the air temperature was recorded hourly during the period from sunset on each survey day to sunrise on the following day (i.e., night‐time air temperature) using data loggers (TRW‐200WR, TOHO, Kanagawa, Japan). The water temperature at each study site was also recorded hourly using data loggers (MX2201, HOBO, Massachusetts, USA) during the same study period. A data logger measuring water temperature was settled at the bottom of each water body and hourly water temperature recordings were made throughout the study period, including the period of adult emergence. In a previous study, a relationship was indicated between daylength exposure during the 5th instar larval stage and the presence or absence of imaginal diapause. When there were differences in larval development patterns of *A. sachalinensis* between alpine and sub‐alpine zones, there were differences in daylength exposure during the 5th instar larval stage. For this reason, daylength data was obtained from the National Astronomical Observatory of Japan (https://eco.mtk.nao.ac.jp/) and correlated between the daylength and period of the 5th instar larval stage.

### 2.3 Data analyses

Head width was measured on larvae collected on each sampling date and study site, and the dataset of all individuals (alpine zone: *n* = 324, sub‐alpine zone: *n* = 911) was used to statistically test for differences in head width of larvae at each stage between alpine and sub‐alpine zones. Data normality was tested using the Shapiro–Wilk normality test. The difference between larval head width of alpine and sub‐alpine zones was analyzed by Mann–Whitney *U*‐test.

Based on the number of days elapsed and the frequency of adult emergence observed using light traps with the starting date as “day 1,” differences in the timing of adult emergence and ovarian development between the alpine and sub‐alpine zones were statistically tested. Data normality was tested using the Shapiro–Wilk normality test. The differences between adult emergence and ovarian development of the alpine and sub‐alpine zones were analyzed using the Mann–Whitney *U*‐test. For all collected adults at each study site, on each sampling date (alpine zone: *n* = 273, sub‐alpine zone: *n* = 596), differences in adult emergence patterns between the alpine and sub‐alpine zones were assessed for the categories of all individuals, males, females, and females undeveloped reproductive organs. Females with Stage A ovarian development were treated as immature females and females with Stage B to D ovarian development were treated as mature females. However, the data about adult emergence collected on June 14 were not used in the analyses as it was only a pilot survey. Therefore, June 21 was set as the start date of the collection of light trap data used for the analyses.

Based on the monthly average of each of the environmental factors (EC, DO, pH, water temperature, air temperature, and canopy openness) measured at each study site, principal component analysis (PCA) was conducted to investigate environmental differences between the alpine and sub‐alpine zones (Table S1).

The Shapiro‐Wilk normality test was performed using the function shapiro.test(), the non‐parametric Mann–Whitney U test was performed using the function wilcox.test(), and PCA was performed using the function prcomp(). These functions were implemented in statistical analysis software R4.2.2 (R Core Team, 2022). PCA plots were plotted using R package ggbiplot (https://github.com/vqv/ggbiplot).

## 3 RESULTS

### 3.1 Comparison of the environmental factors between the alpine and sub‐alpine habitats of *Asynarchus sachalinensis*

**FIGURE 3:**
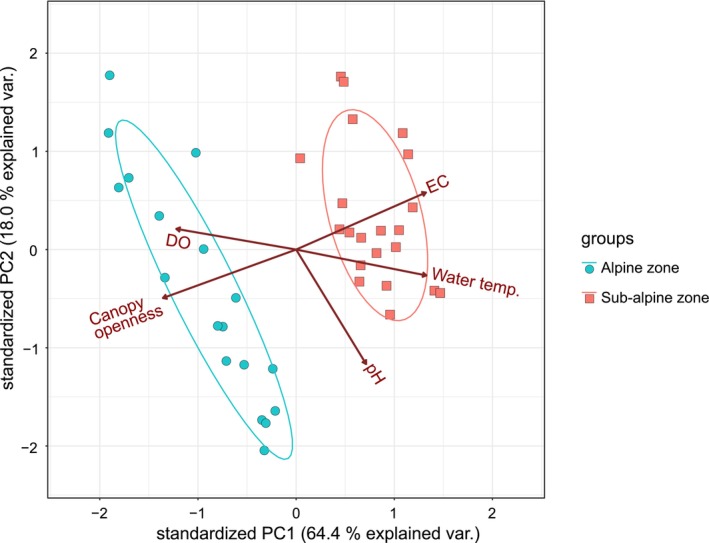
Principal Component Analysis (PCA) of the study sites of *Asynarchus sachalinensis* based on the five environmental factors described in Tables S1 and S2. The points marked with red circles correspond to study sites located within the alpine zone. The points marked with blue squares correspond to study sites located within the sub‐alpine zone. Ellipses indicate the 95% confidence intervals.

**FIGURE 4:**
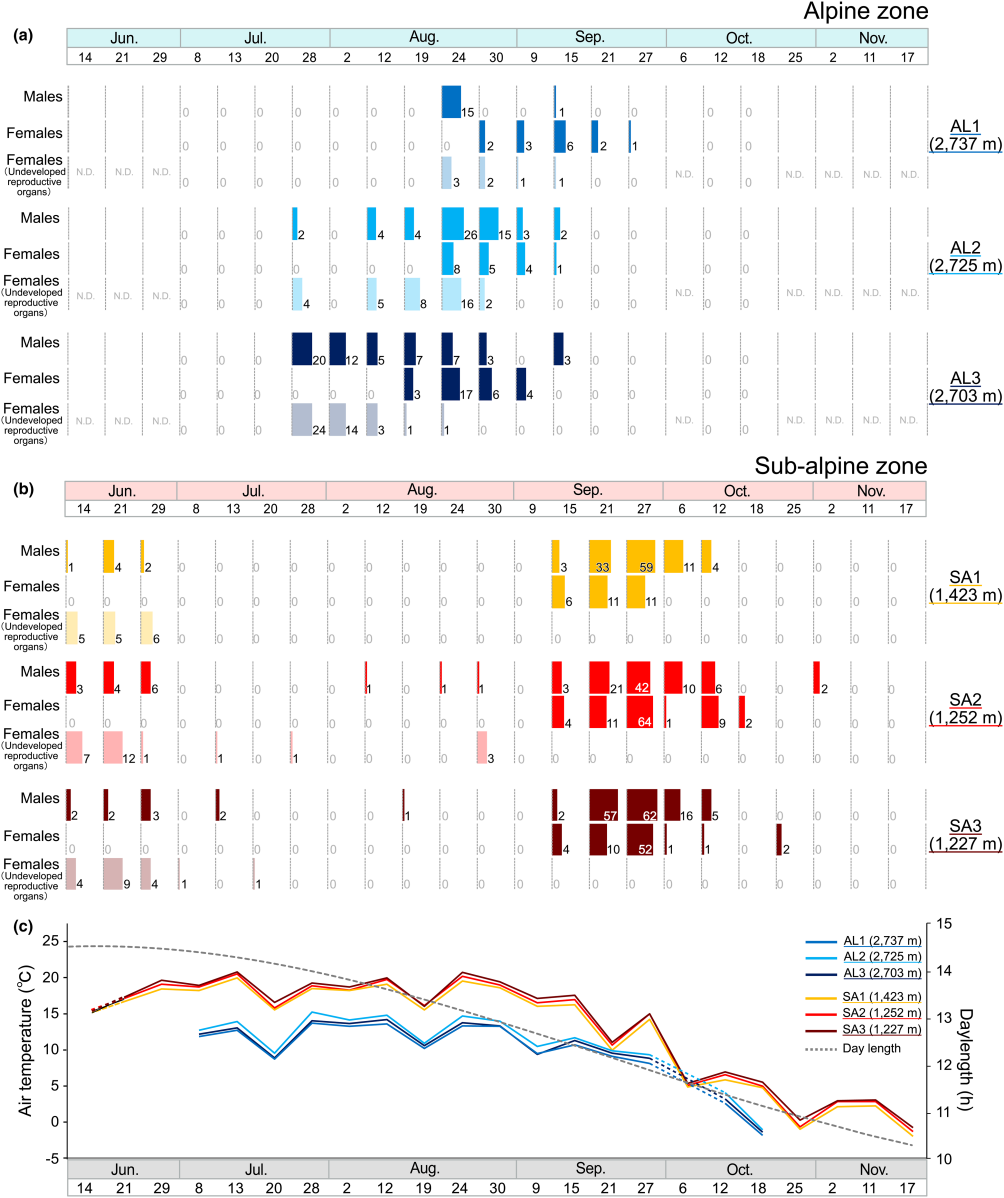
(a, b) The number of adults of *Asynarchus sachalinensis* [Males, Females and Females (Undeveloped reproductive organs)] for each study site in the alpine and sub‐alpine zones. (c) The trends of the average night air temperature at each study site and their respective daylength at Mt. Norikura‐dake.

The results of the PCA of the environmental factors showed that the first accounted for 64.0% of the variance and the second axis 15.5% (Table S2; Figure 3). The ellipsoid 95% probability confidence interval showed that habitats in the alpine and sub‐alpine zones were largely differentiated environmentally (Figure 3). The factor loadings are shown in Table S2. The results showed that the environmental factors most highly contributing to variance of the first axis were canopy openness (−0.500), Water temperature (0.489), EC (0.488), and DO (0.450). The primary factor contributing to in second axis was pH (−0.813) (Table S2). During the adult emergence surveys, night air temperatures tended to be lower in the alpine zone than in the sub‐alpine zone (Figure 4c).

### 3.2 Larval development and the habitat environments of each altitudinal zone

**FIGURE 5:**
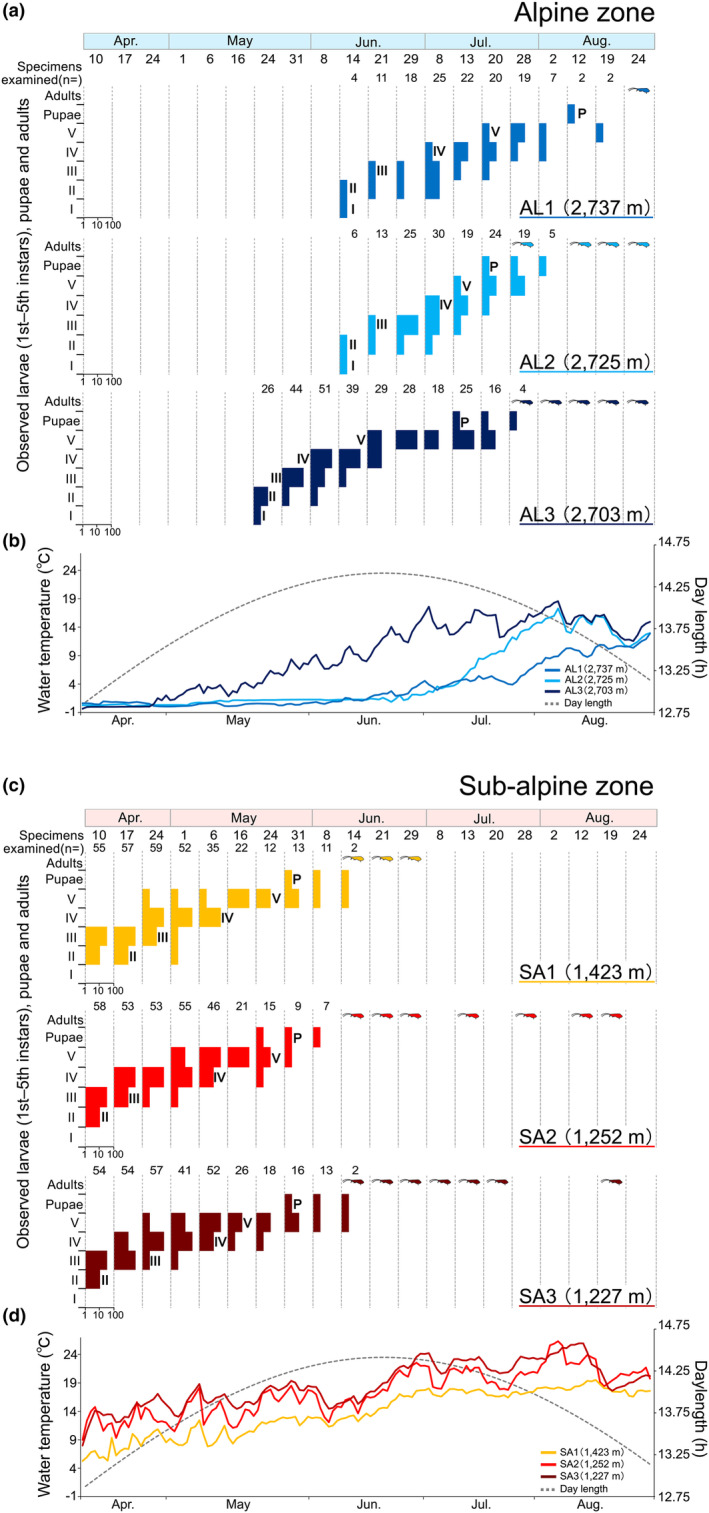
(a, c): The number of specimens at each instar larval stage and pupae of *Asynarchus sachalinensis* observed at each study site in the alpine and sub‐alpine zones. The “caddisfly adults” indicate the period during which adults of *A. sachalinensis* were observed. (b, d): The trends of the average of water temperature at each study site and respective daylength at Mt. Norikura‐dake.

No significant differences were indicated between the head widths of the five instars inhabiting the alpine and sub‐alpine zones (Table 2, Figure S4). The patterns of developmental stage and adult emergence of *A. sachalinensis* did not indicate that different cohorts were present. Thus, *A. sachalinensis* was found to be univoltine in the populations of both altitudinal zones. Additionally, egg masses were observed under stones on land, indicating that *A. sachalinensis* overwinter as eggs or larvae in the egg masses during the winter. Figure 5 shows the differences in the timing of the larval developmental stage and the pattern of water temperature fluctuations in the water bodies at each study site. Regarding the start of larval development, the populations inhabiting the alpine zone started later than the populations inhabiting the sub‐alpine zone, and pupae were observed in the alpine zone more than 1 month later than those in the sub‐alpine zone (Figure 5a,c). In particular, in the water bodies AL1 and AL2 in the alpine zone, there was a tendency for delayed larval development compared to AL3, where a significant rise in water temperature begins earlier in the season. The larvae in AL1 and AL2 had a shorter period in the fifth instar stage compared to AL3 (Figure 5a).

**TABLE 2:** Summary statistics of head width (mm) data for larvae of each instar stage of *Asynarchus sachalinensis* specimens collected in the alpine (AL1–3) and sub‐alpine (SA1–3) zones.

Larval instar	Alpine zone		Sub‐alpine zone		Mann–Whitney *U* test	
	*n*	Head width mean ± SD	*n*	Head width mean ± SD	*W*	*p*‐value
I	14	0.25 ± 0.02	‐	‐	‐	‐
II	72	0.45 ± 0.02	77	0.45 ± 0.03	2390	.147
III	106	0.78 ± 0.03	264	0.78 ± 0.04	12,638	.145
IV	85	1.16 ± 0.05	358	1.16 ± 0.05	16,807	.411
V	47	1.59 ± 0.02	212	1.60 ± 0.06	3680	.052

The date and daylength of first observation of fifth instar larvae at study sites was different in each altitudinal zone; SA1 and SA3 were April 24 and daylength was 13.46 h, while SA2 was May 1 and daylength was 13.62 h in the sub‐alpine zone. In the alpine zone, AL1 was July 28 and daylength was 14.05 h, AL2 was July 13 and daylength was 14.38 h, and AL3 was June 21 and daylength was 14.55 h (Table S3, Figure 5).

### 3.3 Adult emergence and ovarian development

Comparison of adult emergence patterns between the alpine and sub‐alpine zones showed significant differences in the emergence patterns for all categories of all individuals, males, females, and females undeveloped reproductive organs (Table 3, Figure 4c). In the alpine zone, adults were observed intensively from the end of July to the middle of September. At each study site in the alpine zone, the dates when the largest number of adults were observed were August 24 for AL1 and AL2 and July 28 for AL3. In the sub‐alpine zone, adults were observed from mid to late June, but almost no adults were observed in July and August, when air temperatures were higher. Adults were observed again from the middle of September to the middle of October, when air temperatures began to fall. The date when the largest number of adults was observed was September 27 at all study sites in the sub‐alpine zone (Table S3, Figure 4).

**TABLE 3:** Results of the Mann–Whitney *U* test comparing the timing of emergence of adults of *Asynarchus sachalinensis* [all individuals, Males, Females, and Females (Undeveloped reproductive organs)] observed in the alpine and sub‐alpine zones by category.

	Alpine zone		Sub‐alpine zone		Mann–Whitney *U* test	
	*n*	Length of flight period (first date–last date)	*n*	Length of flight period (first date–last date)	*W*	*p*‐value
All individuals	273	Day 23–day 99	596	Day 1–day 135	18,760	<.001
Males	129	Day 38–day 87	363	Day 1–day 135	2,386	<.001
Females	62	Day 23–day 99	189	Day 87–day 127	0	<.001
Females (Undeveloped reproductive organs)	82	Day 38–day 87	44	Day 1–day 71	2,273.5	.012

*Note*: Females (Undeveloped reproductive organs): Developmental stage of ovaries A (Gower, 1963). Females: Developmental stages of ovaries B to D (Gower, 1963). Length of flight period (first date–last date) is recorded as the number of days specimens were collected in the light trap, beginning from the first day which is treated as “day 1.”

**FIGURE 6:**
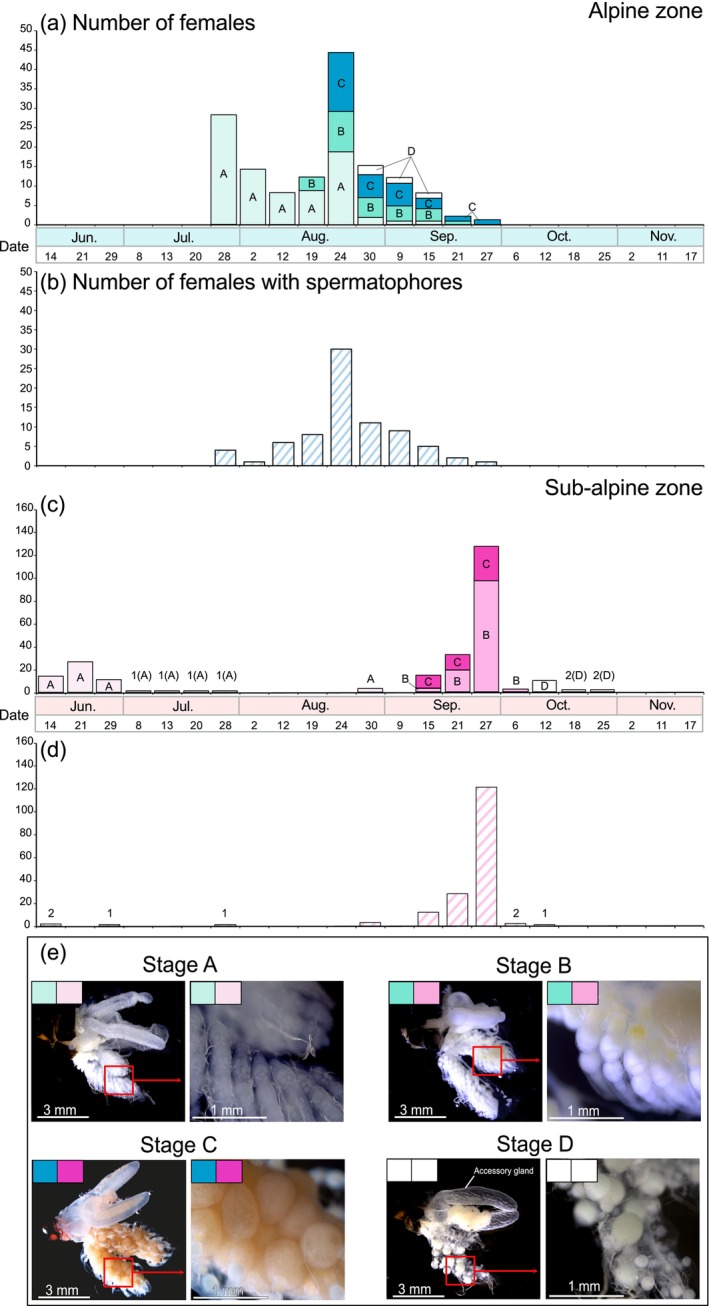
Number of females of *Asynarchus sachalinensis* observed in the alpine and sub‐alpine zones. (a, c): The number of females observed in each altitude zone, classified according to their stage of ovarian development. The colors correspond to the color scheme of their ovarian development classification stages in (e). (b, d): The number of females observed with spermatophores within each altitude zone. (e): Typical stage ontogenetic patterns when ovarian development classified into stages A–D based on Gower (1967).

The pattern of Stage B female emergence was compared between the alpine and sub‐alpine zones. In the alpine zone, the emergence of Stage B females was observed approximately 20 days after the first emergence of adults. On the other hand, in the sub‐alpine zone, the emergence of Stage B females was observed approximately 90 days after the first day of emergence of adults (Figures 4a,b and 6a,c).

Females with spermatophores were observed continuously from mid‐summer to early autumn in the alpine zone. In the sub‐alpine zone, females with spermatophores were observed intermittently in early summer, mid‐summer, and autumn (Figure 6b,d).

## 4 DISCUSSION

### 4.1 The relationships between larval development and environment

In aquatic insects, it is well known that differences in larval development (e.g., ecological differences/life‐history differences) occur due to differences in the altitude of habitats. In particular, habitat temperature is one of the most important environmental factors that alters life‐history patterns (Bonacina et al., 2023; Füreder et al., 2005; Hayashi, 1990; Sweeney, 1984; Vannote & Sweeney, 1980; Ward & Stanford, 1982).

Several studies of limnephilid caddisflies have shown that the larval developmental patterns are determined by water temperature (Gíslason, 1978; Gotceitas & Clifford, 1983; Wissinger et al., 2003). The present survey showed that the season of appearance of early instar larvae of *Asynarchus sachalinensis* differed between the alpine and sub‐alpine zones (alpine zone: early summer season, sub‐alpine zone: spring season). The seasonal timing of larval development and the transition patterns also differed between the alpine and sub‐alpine zones.

Populations of *A. sachalinensis* inhabit lentic waters in alpine zones; *A. sachalinensis* is generally known to inhabit lentic waters and springs (Ito, 2008). Data on water temperature fluctuations indicated that water temperatures increased more slowly in the alpine zone than in the sub‐alpine zone. It was indicated that *A. sachalinensis* exhibited delayed development in the alpine zone waters due to the delayed increase in water temperature.

Additionally, the amount of snow cover varied between the study sites in the alpine zone, indicating that in AL1, where the snow cover was heaviest, the rise in water temperature was delayed and growth was delayed accordingly. However, fifth instar larvae were observed over relatively long periods in sub‐alpine water bodies (SA1–3). In such water bodies, snow cover is relatively lower and snowmelt begins earlier in the season (Figure 5). As such, it was considered probable that factors other than water temperature may be involved in pupation initiation.

In particular, in the studies of the life‐history of caddisflies, differences in daylength are known to be a factor in the induction of pupation and the initiation of larval diapause (Dixon & Wrona, 1992; Elliott, 1968; Malicky, 1981; Tsuruishi, 2003). Life‐history adjustment is based on changes in daylength such that differences in developmental stages between individuals within a population are homogenized, and as a result, reproductive seasons are effectively synchronized (Wiggins, 1996). It is assumed that the daylength to which larvae are exposed affects the timing of emergence and the presence or absence of imaginal diapause, resulting in the synchronization of breeding timing within the population. In fact, our studies indicated that within each altitudinal zone, the emergence of adults was largely synchronous (Figure 4a,b). Therefore, it is indicated that the daylength experienced as larvae was related to their ecological characteristics (i.e., imaginal diapause) as adults.

### 4.2 Habitat environments and imaginal diapause

This occurrence of summer diapause is known as imaginal diapause and occurs in many limnephilid caddisflies (Denis, 1978; Salavert et al., 2008; Svensson, 1972; Wissinger et al., 2003).

In the sub‐alpine zone, the first observation of the adults was in early summer (in June). However, few adults were observed during mid‐summer (in July to August). Of these, a few adults observed shortly after emergence in early summer had not developed ovaries. Most of the individuals observed during autumn (in September to October) had fully developed ovaries (Figure 6c). These results indicated the occurrence of imaginal diapause within sub‐alpine populations of *A. sachalinensis*. To explain the decline in the number of adults in the sub‐alpine zone in mid‐summer, it was assumed that adults were not attracted to the lights due to imaginal diapause. The presence of imaginal diapause in *A. sachalinensis* was already indicated by a previous study of populations in Hokkaido (Ito, 2008). Our study showed similar ecological characteristics of populations inhabiting sub‐alpine zones in Honshu.

On the other hand, in the alpine zone, the emergence of adults was concentrated in mid‐summer (in July to August). Ovarian development of females was observed to be complete earlier than in the sub‐alpine zone, about 30 days after the first adult emergence in the alpine zone. Therefore, imaginal diapause was assumed to be absent or shortened within alpine populations, in contrast to the sub‐alpine populations. The lower temperatures in the alpine zone compared to the sub‐alpine zone may have allowed *A. sachalinensis*, which is ancestrally adapted to cooler environments, to remain active during the summer season. A previous study comparing the life histories of *Limnephilus affinis*, which is a closely related species to *A. sachalinensis*, between populations at different latitudes and altitudes observed that at high latitudes and high altitudes in cooler environments, the adult flight period was concentrated within the summer season (Gíslason, 1978).

Previous studies on limnephilid caddisflies have shown a relationship between imaginal diapause and the daylength during the 5th instar larvae and/or adult stages (Denis, 1978; Novak & Sehnal, 1963). *Asynarchus contumax*, which is a closely related species to *A. sachalinensis*, is distributed at high latitudes of Palaearctic, matures during long‐day conditions, and undergoes no imaginal diapause (Solem, 1983). For *A. sachalinensis*, whose life‐history pattern differs between altitudinal zones, the larval development period likewise differs between the alpine and sub‐alpine zones. Because larval development was at different periods between the alpine and sub‐alpine zones, the daylength of exposure of 5th instar larvae differs even within the same mountain massif. Therefore, differences in the daylength experienced by 5th instar larvae are assumed to be one of the factors influencing the presence or absence of imaginal diapause. This would result in gene flow being highly restricted between these altitudinal zones, strongly supporting the maintenance of genetic differentiation between these zones.

### 4.3 Genetic and ecological differentiation due to altitude

Habitats at high altitudes are often isolated and discontinuous with nearby lowlands (e.g., basins). With such a pattern, the loss of population connectivity restricts chances for gene flow between populations (Knowles, 2001; Mikami et al., 2023; Pauls et al., 2006; Theissinger et al., 2013; Uscanga et al., 2021).

Phylogenetic analyses based on sequencing of the mtDNA COI region conducted in previous studies showed differing genetic structures between alpine and subalpine *Asynarchus sachalinensis* populations. In particular, a single unique mountain massif‐specific haplotype was detected in the alpine populations, which was not found among other populations (Suzuki et al., 2024). Therefore, such alpine populations are isolated and genetic differentiation has occurred (Suzuki et al., 2024). Not only geographical isolation, but also differences in life history between alpine and sub‐alpine zones and differences in breeding season would lead to reproductive isolation. In our study, the pattern of occurrence of females with spermatophores in their bursa is different between the alpine and sub‐alpine zones. The females with spermatophores indicated that they had mated immediately after emergence within each altitudinal zone. Thus, showing that the mating seasons correspondingly differ between the altitudinal zones.

No previous studies have provided data on the flight ability of *A. sachalinensis*. However, it has been observed that *Chaetopterygopsis maclachlani* (Limnephilidae), which inhabits the European Alps, has very low flight ability (Lehrian et al., 2010; Wagner, 1992). Phylogenetic analyses conducted based on sensitive molecular markers (e.g., genome‐wide SNPs and/or microsatellite markers) will show that there is limited dispersal and gene flow between altitudinal zones. Therefore, the knowledge obtained from our other study will provide an understanding of the relationship between genetic and ecological differentiation between altitudinal zones.

## AUTHOR CONTRIBUTIONS

**Hirohisa Suzuki:** Conceptualization (equal); formal analysis (equal); writing – original draft (equal). **Masaki Takenaka:** Conceptualization (equal); formal analysis (equal); writing – review and editing (equal). **Koji Tojo:** Conceptualization (equal); formal analysis (equal); project administration (lead); writing – review and editing (lead).

## CONFLICT OF INTEREST STATEMENT

The authors declare that they have no known competing financial interests or personal relationships that could have appeared to influence the results reported in this paper.

## Supporting information


Figure S1.
Figure S2.
Figure S3.
Figure S4.
Table S1.
Table S2.
Table S3.

## Data Availability

The authors confirm that the data supporting the findings of this study are available within the article and in its supplementary data.
