# Supplementary material for: Variations in the phenological patterns of a caddisfly inhabiting the same mountain massifs: Life‐history differences in different altitudinal zones
Source: Ecol Evol. 2024 Jun 6;14(6):e11428. doi: 10.1002/ece3.11428 (PMC11157149; doi:10.1002/ece3.11428)
Supplement: Supplementary file 1 — Figure S1. Figure S2. Figure S3. Figure S4. Table S1. Table S2. Table S3. [file ECE3-14-e11428-s001.docx]

| **Table S1.** Environmental factors of each study site used for principal component analysis (PCA). The monthly data used is averaged | | | | | | |
| --- | --- | --- | --- | --- | --- | --- |
|  |  |  |  |  |  |  |
| Locality  ID | Month | Electrical  Conductivity (EC: μS) | Dessoved  Oxigen (DO: mg/L) | pH | Canopy  openness rate (%) | Water  temperature (℃) |
| AL. 1 | May | 7.410 | 11.813 | 5.85 | 92.82 | 0.40 |
|  | Jun. | 6.928 | 11.515 | 5.75 | 91.78 | 2.18 |
|  | Jul. | 6.828 | 10.748 | 5.85 | 92.37 | 5.00 |
|  | Aug. | 4.914 | 8.964 | 6.35 | 92.02 | 13.28 |
|  | Sep. | 5.910 | 8.923 | 6.80 | 91.57 | 13.90 |
|  | Oct. | 6.217 | 9.023 | 6.25 | 91.07 | 8.40 |
| AL. 2 | May | 4.840 | 11.680 | 5.54 | 90.72 | 0.48 |
|  | Jun. | 5.415 | 11.713 | 5.21 | 91.33 | 3.33 |
|  | Jul. | 5.233 | 9.328 | 5.32 | 91.38 | 11.58 |
|  | Aug. | 6.666 | 8.418 | 6.29 | 92.62 | 17.38 |
|  | Sep. | 6.120 | 8.785 | 6.64 | 90.55 | 14.38 |
|  | Oct. | 6.217 | 9.023 | 6.25 | 91.07 | 8.40 |
| AL. 3 | May | 4.528 | 10.858 | 6.14 | 92.80 | 5.30 |
|  | Jun. | 3.605 | 9.953 | 5.82 | 91.97 | 12.70 |
|  | Jul. | 4.398 | 9.060 | 5.98 | 92.27 | 14.85 |
|  | Aug. | 4.976 | 8.450 | 6.50 | 92.50 | 16.88 |
|  | Sep. | 5.743 | 8.865 | 6.63 | 91.48 | 14.23 |
|  | Oct. | 6.067 | 8.770 | 6.41 | 94.23 | 7.83 |
| SA. 1 | Apr. | 44.300 | 7.416 | 5.49 | 40.65 | 14.00 |
|  | May | 45.020 | 7.390 | 5.66 | 43.40 | 15.22 |
|  | Jun. | 44.125 | 8.313 | 5.50 | 36.05 | 16.90 |
|  | Jul. | 45.975 | 7.618 | 5.77 | 19.77 | 22.10 |
|  | Aug. | 44.876 | 6.880 | 5.86 | 18.80 | 19.76 |
|  | Sep. | 40.680 | 8.258 | 6.39 | 28.68 | 17.73 |
|  | Oct. | 41.225 | 8.200 | 6.35 | 35.83 | 10.43 |
|  | Nov. | 41.567 | 9.927 | 6.53 | 52.02 | 6.30 |
| SA. 2 | Apr. | 48.787 | 9.649 | 6.47 | 31.48 | 17.50 |
|  | May | 43.792 | 9.356 | 6.57 | 29.00 | 16.46 |
|  | Jun. | 43.725 | 8.580 | 6.43 | 17.83 | 20.15 |
|  | Jul. | 46.175 | 7.818 | 6.56 | 20.00 | 24.20 |
|  | Aug. | 44.320 | 7.720 | 6.54 | 16.62 | 25.10 |
|  | Sep. | 43.250 | 8.988 | 6.80 | 21.98 | 18.70 |
|  | Oct. | 45.250 | 9.148 | 6.54 | 19.82 | 11.13 |
|  | Nov. | 48.767 | 10.120 | 6.61 | 31.92 | 6.33 |
| SA. 3 | Apr. | 49.400 | 10.845 | 6.26 | 30.50 | 10.40 |
|  | May | 45.016 | 9.166 | 6.31 | 29.22 | 12.94 |
|  | Jun. | 44.225 | 8.363 | 6.34 | 25.04 | 17.58 |
|  | Jul. | 50.825 | 7.610 | 6.24 | 20.00 | 19.50 |
|  | Aug. | 47.800 | 7.728 | 6.40 | 17.90 | 15.98 |
|  | Sep. | 47.325 | 8.795 | 6.77 | 16.35 | 15.08 |
|  | Oct. | 45.650 | 8.920 | 6.74 | 25.38 | 11.88 |
|  | Nov. | 52.767 | 10.903 | 6.71 | 34.72 | 6.17 |

| **Table S2.** Principal component analysis (PCA) loaded scores of environmental factors for PC1 to PC5 were used to estimate variance between the study sites of the alpine (AL1–3) and sub-alpine (SA1–3) zones | | | | | |  |
| --- | --- | --- | --- | --- | --- | --- |
| Environmental factors | PC1 | PC2 | PC3 | PC4 | PC5 | |
| Electrical Conductivity (EC) | 0.488 | 0.405 | 0.331 | 0.116 | 0.689 | |
| Dessoved Oxigen (DO) | -0.450 | 0.147 | 0.611 | -0.633 | 0.045 | |
| pH | 0.263 | -0.813 | 0.505 | 0.114 | 0.030 | |
| Canopy openness rate | -0.500 | -0.344 | -0.337 | 0.002 | 0.719 | |
| Water temperature | 0.489 | -0.184 | -0.385 | -0.757 | 0.075 | |
|  |  |  |  |  |  | |
| Proportion of Variance (%) | **0.644** | **0.180** | **0.140** | **0.033** | **0.003** | |

| **Table S3.** The date and daylength observed of the first five instar larvae specimens collected at each study site. The date and daylength observed of adult specimens collected at each site for each of the largest number of adult specimens collected | | | | | |
| --- | --- | --- | --- | --- | --- |
|  | 5th instar larvae first observed | |  | Largest number of adults observed | |
| Locality ID | Date | Day length |  | Date | Day length |
| AL. 1 | Jul. 28 | 14.05 |  | Aug. 24 | 13.22 |
| AL. 2 | Jul. 13 | 14.38 |  | Aug. 24 | 13.22 |
| AL. 3 | Jun. 21 | 14.55 |  | Jul. 28 | 14.05 |
| SA. 1 | Apr. 24 | 13.38 |  | Sep. 27 | 12.00 |
| SA. 2 | May 1 | 13.62 |  | Sep. 27 | 12.00 |
| SA. 3 | Apr. 24 | 13.38 |  | Sep. 27 | 12.00 |


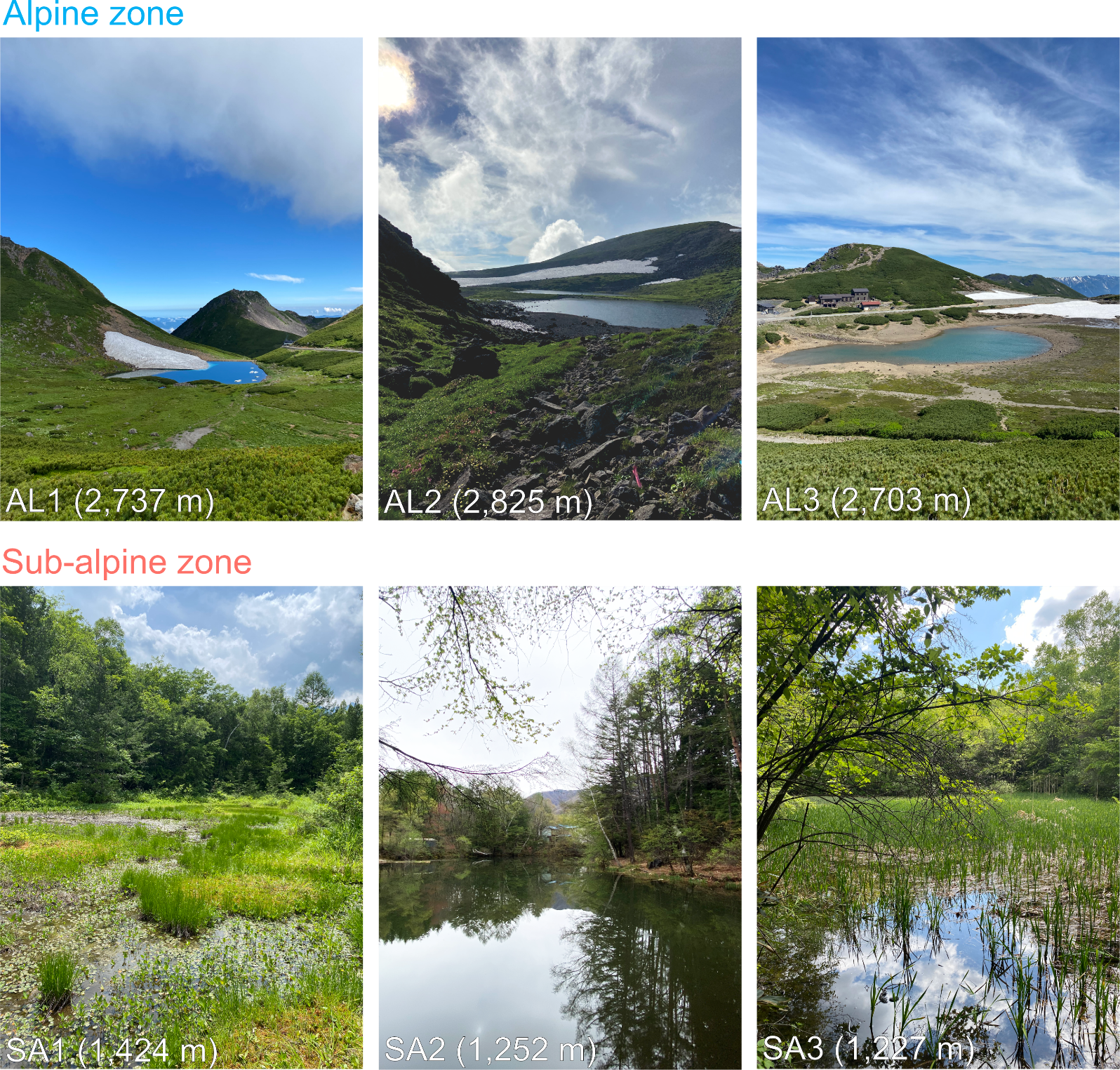
**Figure S1.** The landscapes around the water bodies of the study sites. AL1–3 sites were located in the alpine zone, and SA1–3 sites were located in the sub-alpine zone.


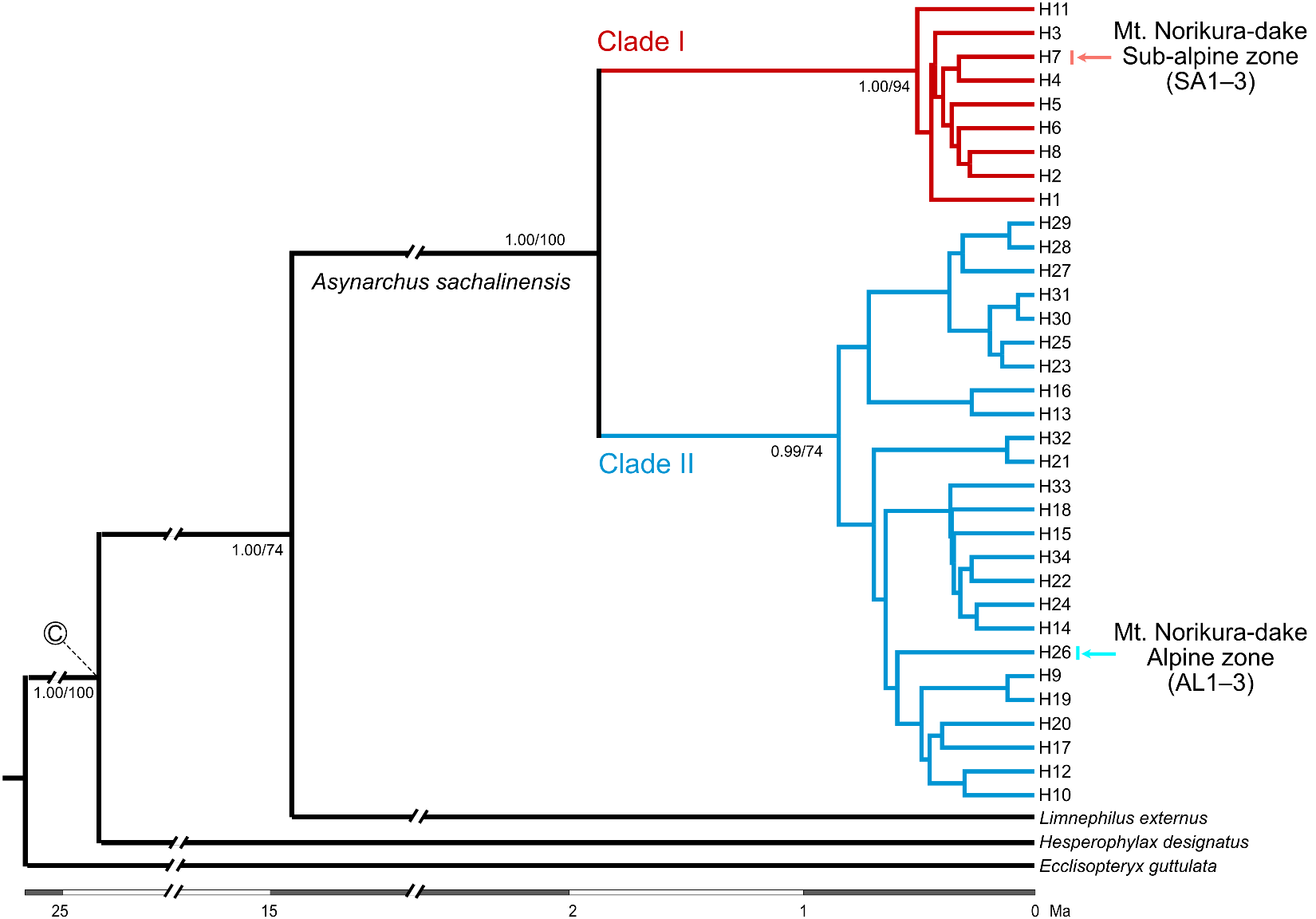


**Figure S2.** The cladogram was partially modified from Suzuki et al. (2024). Phylogenetic analyses were performed using two combined sequences of the mtDNA COI (648 bp) and 16S rRNA (417 bp) regions of *Asynarchus sachalinensis*. The numbers at major nodes indicate the Bayesian posterior probabilities and the ML bootstrap values. Detailed information about the dataset and methods of genetic analyses are described in Suzuki et al. (2024). The haplotype H7 was detected in specimens collected from the sub-alpine zone (SA1–3), while the haplotype H26 was detected in specimens collected from the alpine zone (AL1–3).


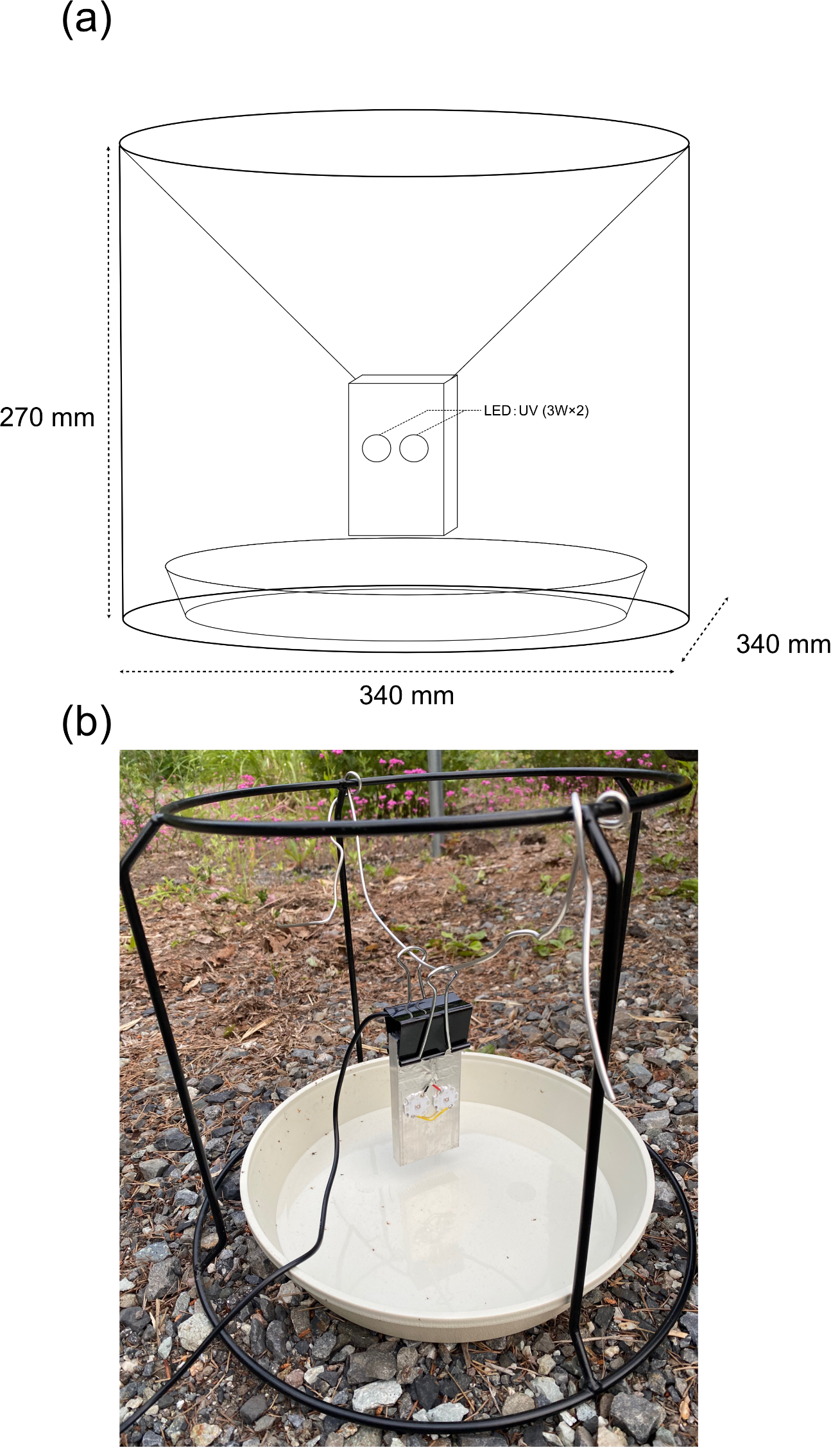


**Figure S3.** (a) A schematic figure of the light trap. (b) A photograph of the light trap after set up.


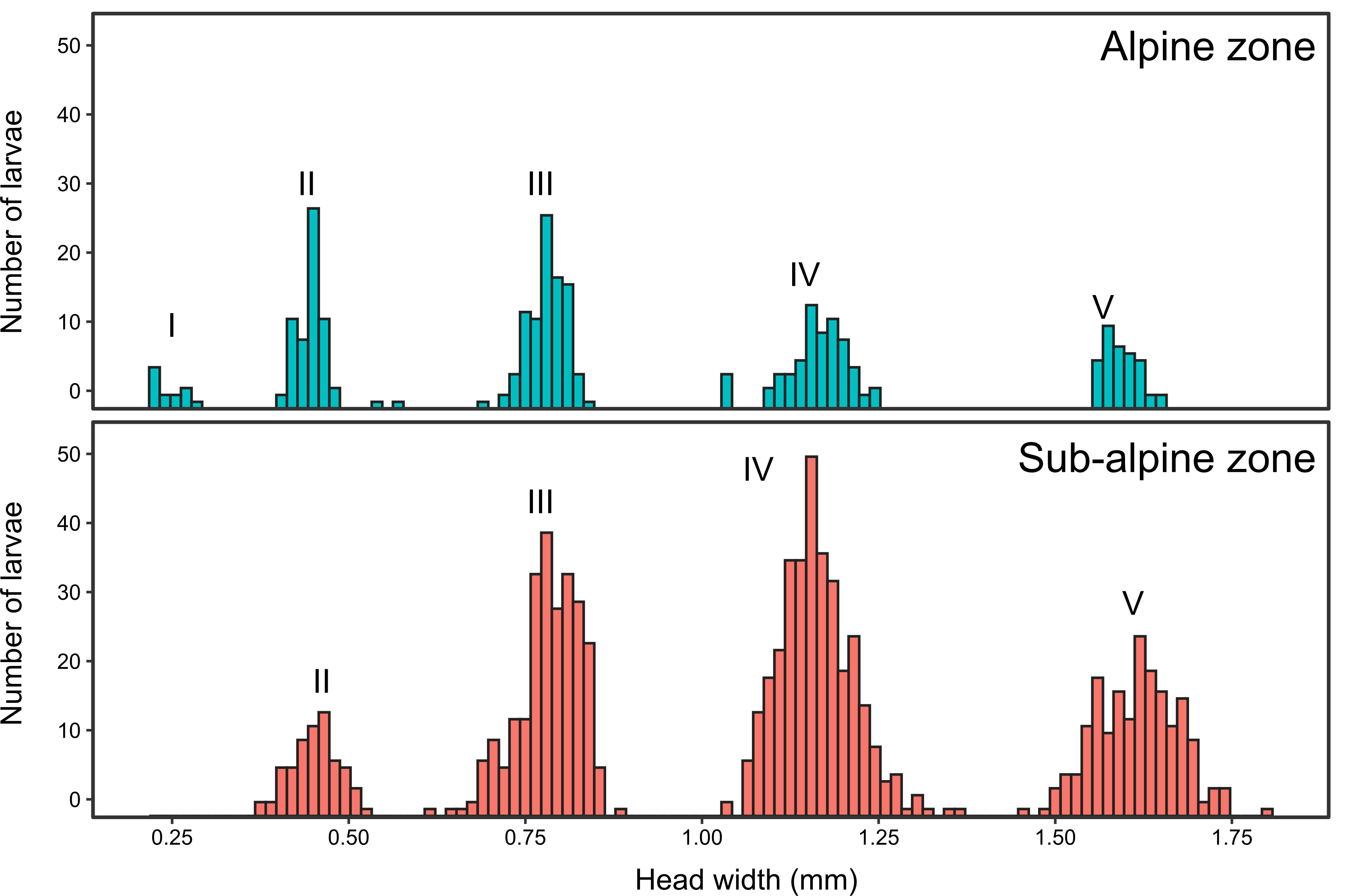


**Figure S4.** Histograms of larval head widths as observed in the alpine and sub-alpine zones.
